# Supplementary material for: Cell-Specific Effects of Insulin in a Murine Model of Restenosis Under Insulin-Sensitive and Insulin-Resistant Conditions
Source: Cells. 2024 Aug 20;13(16):1387. doi: 10.3390/cells13161387 (PMC11352246; doi:10.3390/cells13161387)
Supplement: Supplementary file 1 [file cells-13-01387-s001.zip › cells-3031654-supplementary.pdf]

Supplementary Table S1. Insulin Tolerance Test (ITT) Area Under the Curve (AUC) values for tamoxifen-treated endothelial cell (EC)- and smooth muscle cell (SMC)-specific insulin receptor (IR) deficient ($IR^{f/f}$-Cre(+)) and control ($IR^{f/f}$-Cre(-)) mice. Data are expressed as mean ± SEM. LFD: low fat diet. HFSD: high fat, high sucrose diet. Parametric or non-parametric tests were applied according to the results of normality tests. N.S. One-way ANOVA followed by Tukey test. *n*>6.

	**LFD, Vehicle**		**LFD, Insulin**		**HFSD, Vehicle**		**HFSD, Insulin**	
	$IR^{f/f}$-Cre(-)	*$IR^{f/f}$-Cre(+)*	*$IR^{f/f}$-Cre(-)*	*$IR^{f/f}$-Cre(+)*	*$IR^{f/f}$-Cre(-)*	*$IR^{f/f}$-Cre(+)*	*$IR^{f/f}$-Cre(-)*	*$IR^{f/f}$-Cre(+)*
**EC-IR deficient and controls**	994 ± 67	860 ± 34	984 ± 69	765 ± 185	1690 ± 73	1541 ± 82	1524 ± 62	1588 ± 144
**SMC-IR deficient and controls**	994 ± 67	1196 ± 96	984 ± 69	975 ± 95	1690 ± 73	1600 ± 40	1524 ± 62	1589 ± 70

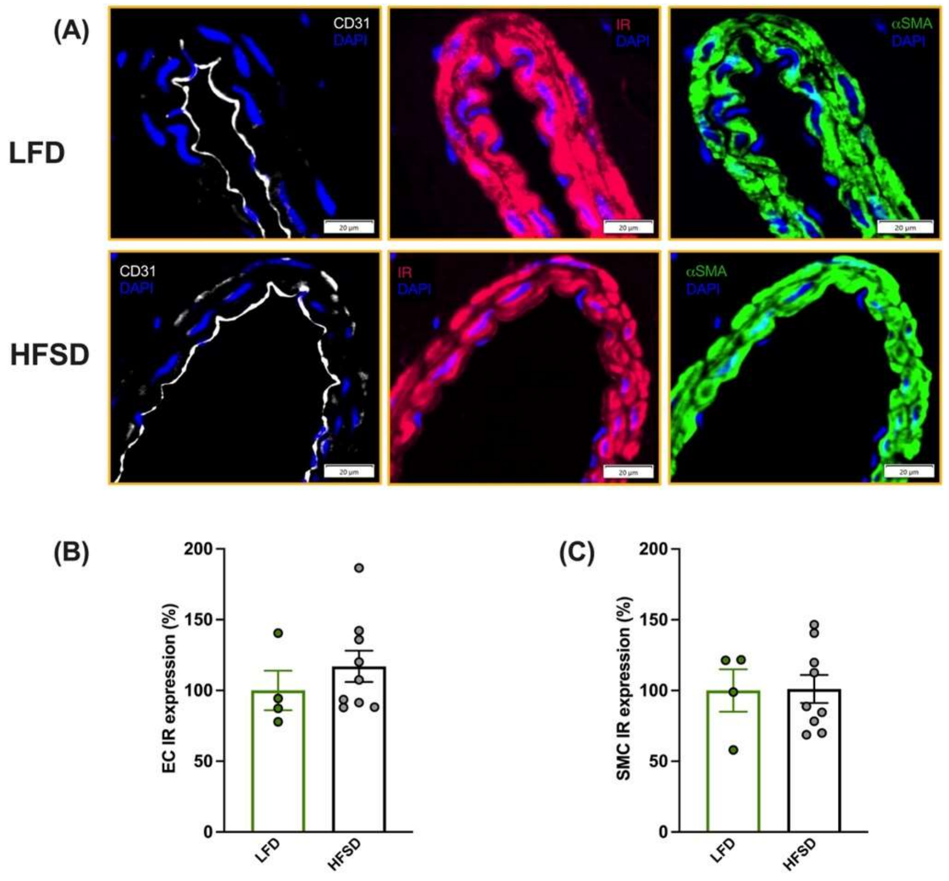

Supplementary Figure S1. Immunofluorescence analysis of the expression of insulin receptor (IR) in endothelial cells (ECs) and vascular smooth muscle cells (SMCs) of low fat diet (LFD)-fed -or high fat, high sucrose diet (HFSD)-fed tamoxifen-treated *$IR^{f/f}$-Cre(−)* control mice. Uninjured femoral arteries were collected to assess IR expression in ECs and SMCs by immunofluorescence. **(A)**: Representative immunofluorescence images of femoral arteries stained for IR, CD31, and α-SMA in LFD-or HFSD-fed mice (x60). **(B)**: Expression of IR in endothelial cells (percentage of the average in LFD). **(C)**: Expression of IR in smooth muscle cells (percentage of the average in LFD). Empty green bars, LFD; empty black bars, HFSD. LFD, *n* = 4; HFSD, *n*=9. Data are expressed as mean ± SEM. Parametric or non-parametric tests were applied according to the results of normality tests. N.S. Two-tailed unpaired t-test.

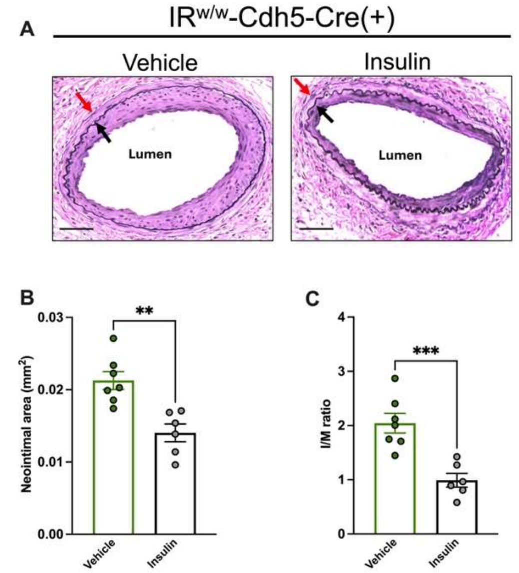

Supplementary Figure S2. Insulin treatment reduces neointimal growth in tamoxifen-treated low fat diet (LFD)-fed *IR$^{w/w}$-Cdh5-Cre(+)* control male mice. Injured femoral arteries were collected 28 days after wire injury. **(A):** Representative images of femoral arteries stained with Elastin Van Gieson (EVG) (x 200). Black arrows indicate internal elastic lamina, and red arrows indicate external elastic lamina. **(B):** Neointimal area. **(C):** Intima to media (I/M) ratio. Empty green bars, vehicle; empty black bars, insulin. Scale bar: 50μm. *IR$^{w/w}$-Cdh5-Cre(+)*: Vehicle , *n*= 7; Insulin, *n*= 6. Data are expressed as mean ± SEM. Parametric or non-parametric tests were applied according to the results of normality tests. Two-tailed unpaired t-test: ** p<0.01; *** p<0.001.

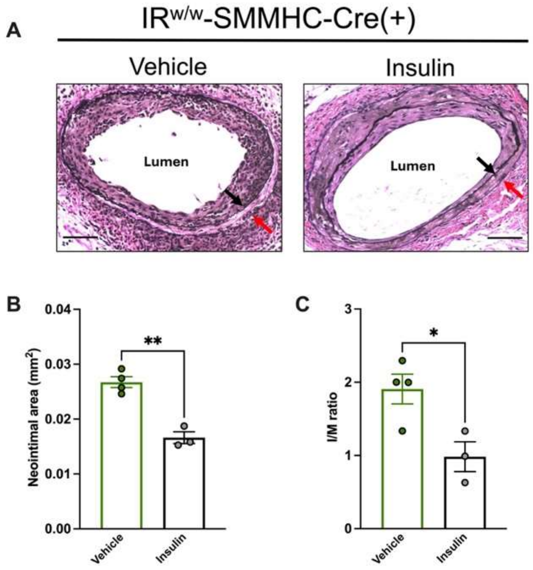

Supplementary Figure S3. Insulin treatment reduces neointimal growth in tamoxifen-treated low fat diet (LFD)-fed *IR$^{w/w}$-SMMHC-Cre(+)* control male mice. Injured femoral arteries were collected 28 days after wire injury. **(A):** Representative images of femoral arteries stained with Elastin van Gieson (EVG) (x 200). Black arrows indicate internal elastic lamina, and red arrows indicate external elastic lamina. **(B):** Neointimal area. **(C):** Intima to media (I/M) ratio. Empty green bars, vehicle; empty black bars, insulin. Scale bar: 50μm. *IR$^{w/w}$-SMMHC-Cre(+)*: Vehicle, *n*= 4; Insulin, *n*= 3. Data are expressed as mean ± SEM. Parametric or non-parametric tests were applied according to the results of normality tests. Two-tailed unpaired t-test: * p<0.05; ** p<0.01.

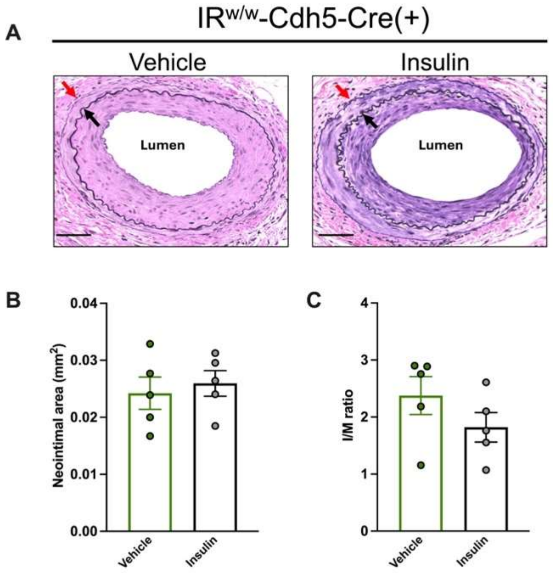

Supplementary Figure S4. Insulin treatment failed to reduce neointimal growth in tamoxifen-treated high fat, high sucrose (HFSD)-fed *IR$^{w/w}$-Cdh5-Cre(+)* control male mice. Injured femoral arteries were collected 28 days after wire injury. **(A):** Representative images of femoral arteries stained with Elastin Van Gieson (EVG) (x 200). Black arrows indicate internal elastic lamina, and red arrows indicate external elastic lamina. **(B):** Neointimal area. **(C):** Intima to media (I/M) ratio. Empty green bars, vehicle; empty black bars, insulin. Scale bar: 50μm. IR$^{w/w}$-Cdh5-Cre(+): Vehicle, *n*= 5; Insulin, *n*= 5. Data are expressed as mean ± SEM. Parametric or non-parametric tests were applied according to the results of normality tests. N.S. Two-tailed unpaired t-test.

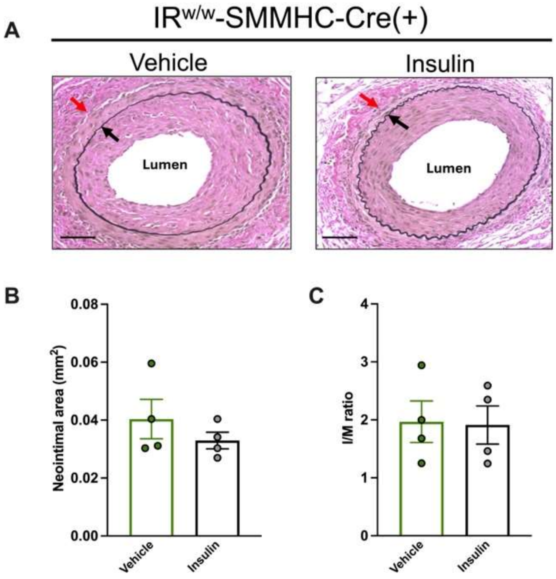

Supplementary Figure S5. Insulin treatment failed to reduce neointimal growth in tamoxifen-treated high fat, high sucrose (HFSD)-fed *IR$^{w/w}$-SMMHC-Cre(+)* control male mice. Injured femoral arteries were collected 28 days after wire injury. **(A):** Representative images of femoral arteries stained with Elastin Van Gieson (EVG) (x 200). Black arrows indicate internal elastic lamina, and red arrows indicate external elastic lamina. **(B):** Neointimal area. **(C):** Intima to media (I/M) ratio. Empty green bars, vehicle; empty black bars, insulin. Scale bar: 50μm. *IR$^{w/w}$-SMMHC-Cre(+)*: Vehicle, *n*= 4; Insulin, *n*= 4. Data are expressed as mean ± SEM. Parametric or non-parametric tests were applied according to the results of normality tests. N.S. Two-tailed unpaired t-test.
